# Extracellular Vesicle-like Associated microRNAs in Monofloral Honeys: Molecular Characterization and Functional Pathways

**DOI:** 10.3390/ijms27125297

**Published:** 2026-06-11

**Authors:** Diana Marisol Abrego-Guandique, Silvia Nuzzo, Olubukunmi Amos Ilori, Ilaria Leone, Mario Zanfardino, Enrico Gallo, Paola Tucci, Filippo Luciani, Maria Cristina Caroleo, Roberto Cannataro, Erika Cione

**Affiliations:** 1 Department of Health Sciences, University of Magna Graecia, 88100 Catanzaro, Italy; dianamarisol.abregoguandique@unicz.it (D.M.A.-G.); mariacristina.caroleo@unicz.it (M.C.C.); 2 IRCCS Synlab SDN, Via Emanuele Gianturco 113, 80143 Naples, Italy; silvia.nuzzo@synlab.it (S.N.); ilaria.leone@synlab.it (I.L.); mario.zanfardino@synlab.it (M.Z.); enrico.gallo@synlab.it (E.G.); 3 Department of Pharmacy, Health and Nutritional Sciences, University of Calabria, 87036 Rende, Italy; olubukunmiamos.ilori@unical.it (O.A.I.); paola.tucci@unical.it (P.T.); 4 Galascreen Laboratories, University of Calabria, 87036 Rende, Italy; filippoluciani@gmail.com (F.L.); r.cannataro@gmail.com (R.C.); 5 Research Division, Dynamical Business & Science Society, DBSS International SAS, Bogotá 110311, Colombia

**Keywords:** microRNA, functional food, honey, extracellular vesicles, biomarkers, bioinformatics

## Abstract

Recent studies have identified microRNAs (miRNAs) in honey, opening a new and promising area of nutrition research. In this view, pasteurized and unpasteurized samples of Eucalyptus, Orange Blossom, Chestnut, and Sulla honeys were analyzed using manual and semi-automated RNA extraction methods. Semi-automated extraction yielded significantly higher RNA amounts than manual methods, while pasteurization selectively affected miRNA presence, depending on the type of honey. The panel of conserved miRNAs monitored was let-7a-5p, miR-1-3p, miR-7-5p, miR-10a-5p, miR-33a-5p, miR-34a-5p, miR-92a-3p, miR-125b-5p and miR-133a-3p, across honey varieties and in their extracellular vesicles with structures approximately 200 nm in diameter that retain four miRNAs in all honey types, miR-1-3p, miR-34a-5p, miR-92a-3p, and miR-133a-3p. Bioinformatic analyses of validated miRNA targets revealed enrichment in pathways related to cytoskeletal organization, transcriptional regulation, protein stability, and immune system processes, with Reactome categories clustering around signal transduction, protein metabolism, and immune interactions. Cell–type–specific enrichment suggested that gastric isthmus progenitor cells, stromal cells, and immune subsets could be potential targets, implying roles in epithelial renewal, immune modulation, and wound healing. Overall, these findings enhance our understanding of honey as a source of conserved miRNAs in extracellular vesicles, highlighting its potential as a natural carrier that protects miRNAs from degradation. This study offers new insights into the health-promoting properties of honey, warranting further preclinical studies.

## 1. Introduction

Honey, a natural food produced by bees (*Apis mellifera* and *Meliponinae*), has been valued for its nutritional and medicinal properties throughout human history [1,2,3]. Its composition, primarily consisting of fructose, glucose, water, organic acids, enzymes, amino acids, minerals, vitamins, and aromatic compounds, varies significantly depending on factors such as floral source, season, processing methods, storage conditions, and geographical origin [4,5,6]. Italy, particularly the Calabrian region, is renowned for its diverse and high-quality honey varieties, which are influenced by the region’s unique flora and climatic conditions [7]. Beyond its high sugar content, honey is rich in bioactive compounds, including carotenoids, phenolic acids, and flavonoids, contributing to its antioxidant, anti-inflammatory, antiproliferative, and wound-healing properties [8,9,10,11,12]. In recent years, the total RNA fraction of honey, tiny non-coding RNAs, has garnered increasing attention as a potential source of bioactive molecules [13]. The detection of microRNAs (miRNAs, miRs) in food matrices [14] and reports suggesting that some dietary miRNAs may resist digestion and be absorbed from dietary sources [15] have stimulated interest in profiling miRNAs in various food products. However, the concept of dietary miRNA uptake and cross-kingdom miRNA transfer remains controversial. While most studies have focused on plant-derived miRNAs [16], honey was first investigated as a potential source of miRNAs by Masood and co-workers [17]. Subsequent research has explored the presence of plant miRNAs in honey, raising the possibility of cross-genera miRNA transfer [16]. Nevertheless, controlled pollination studies have demonstrated that the transfer of plant miRNAs into honeybees is minimal [17]. In our previous work, we identified miRNAs in polyfloral honey. We demonstrated their stability during gastrointestinal digestion, suggesting that honey-derived miRNAs may survive the digestive process and could potentially contribute to biological effects [18]. Extracellular vesicles (EVs) have emerged as critical mediators of intercellular communication, playing a key role in stabilizing and delivering miRNAs in biological fluids even during infections [19]. Recent studies have identified EV-like in honey, suggesting that these vesicles may act as natural carriers that protect miRNAs from degradation and enhance their bioactivity upon ingestion, as is true for milk [18,20,21]. Building on these insights, this study aims to evaluate the presence of conserved miRNAs in selected honey varieties from the Calabria region. We consider the effects of honey processing methods, such as pasteurization and RNA extraction techniques, to better understand how these factors influence miRNA stability and yield. Additionally, we isolated EV-like detecting miRNAs within these vesicles, supporting their possible role in miRNA stabilization. Then, we employ bioinformatics analyses to investigate the potential targets and pathways associated with the honey-miRNAs, providing a deeper understanding of their biological role. By integrating these approaches, we aim to elucidate how miRNAs in honey contribute to their possible biological roles, particularly in immune modulation and wound healing.

## 2. Results

### 2.1. Effects of Extraction Methods and Pasteurization on the RNA Yield and Purity

A comparative analysis of total RNA yield and purity was conducted for four monofloral honey types (Eucalyptus, Orange Blossom, Chestnut, and Sulla) using manual and semi-automated RNA extraction methods (Table 1). The semi-automated method consistently demonstrated higher total RNA yields, with concentrations ranging from 1.61 to 3.46 µg RNA/g of honey, compared to the manual method, which yielded 0.45 to 0.95 µg RNA/g. Among the honey types, Chestnut and Sulla exhibited the highest RNA concentrations. Although the semi-automated method improved yield, it did not significantly enhance RNA purity, as measured by absorbance ratios (A260/A280). However, it provided greater consistency in RNA purity across pasteurized and unpasteurized honey pairs. Notably, unpasteurized honey samples generally yielded higher RNA concentrations than their pasteurized counterparts, except for the Sulla variety, which showed a similar trend in both extraction methods. Overall, the semi-automated method proved superior in terms of RNA yield.

### 2.2. Alignment of Human miRNA with Apis mellifera Homolog

Sequence alignment analysis revealed a notable degree of evolutionary conservation between several human miRNAs and their homologous sequences in *Apis mellifera.* Multiple human miRNAs, including let-7a-5p, miR-1-3p, miR-10a-5p, miR-33a-5p, and miR-133a-3p, exhibited 100% identity with their corresponding honeybee sequences, with query coverages ranging from approximately 85% to over 95%. These alignments were gap-free, indicating a strong sequence match [22,23]. Other miRNAs, such as miR-92a-3p and miR-125a-5p, demonstrated high but slightly lower identity values (95% and 86.36%, respectively), with query coverages above 90% and no gaps in most cases. miR-34a-5p showed 90% identity and 86.36% query coverage, while miR-7-5p, despite a 100% identity, aligned with a considerably lower target coverage of 25.29%, suggesting a more limited conserved region (Figure S1).

### 2.3. Conserved miRNAs in Monofloral Honey Varieties

Of the nine miRNAs let-7a-5p, miR-1-3p, miR-7-5p, miR-10a-5p, miR-33a-5p, miR-34a-5p, miR-92a-3p, miR-125b-5p, and miR-133a-3p measured by qPCR in the different honey varieties, only four (miR-1-3p, miR-92a-3p, miR-133a-3p, and miR-34a-5p) were consistently detected in four monofloral honey varieties (Eucalyptus, Orange Blossom, Chestnut, and Sulla) under both unpasteurized and pasteurized conditions. Furthermore, data from manual and semi-automated extraction methods indicate a significant improvement in the detection of miR-1-3p in pasteurized Eucalyptus, Orange Blossom, and Chestnut honeys compared to manual extraction (*p* < 0.05), while no differences were observed in Sulla. The miR-33a-5p showed higher detection with the semi-automated method in Eucalyptus and Chestnut honeys (*p* < 0.05), but not in Orange Blossom or Sulla. The levels of miR-34a-5p were unaffected by the extraction method or pasteurization in all honeys. For miR-92a-3p, no significant differences were found in Eucalyptus, whereas Orange Blossom, Chestnut, and Sulla honeys consistently showed enhanced detection with semi-automated extraction regardless of pasteurization (*p* < 0.05). Finally, miR-133a-3p was significantly more detectable by the semi-automated method in Eucalyptus and pasteurized Orange Blossom honeys (*p* < 0.05), with no significant changes observed in Chestnut and Sulla. Overall, semi-automated extraction enhanced miRNA detection in most honey varieties, particularly in Orange Blossom and Chestnut, whereas pasteurization alone had no consistent effect, as shown in Supplementary Figure S2.

### 2.4. Isolation and Characterization of Honey Extracellular Vesicles-like and miRNA

**Figure 1:**
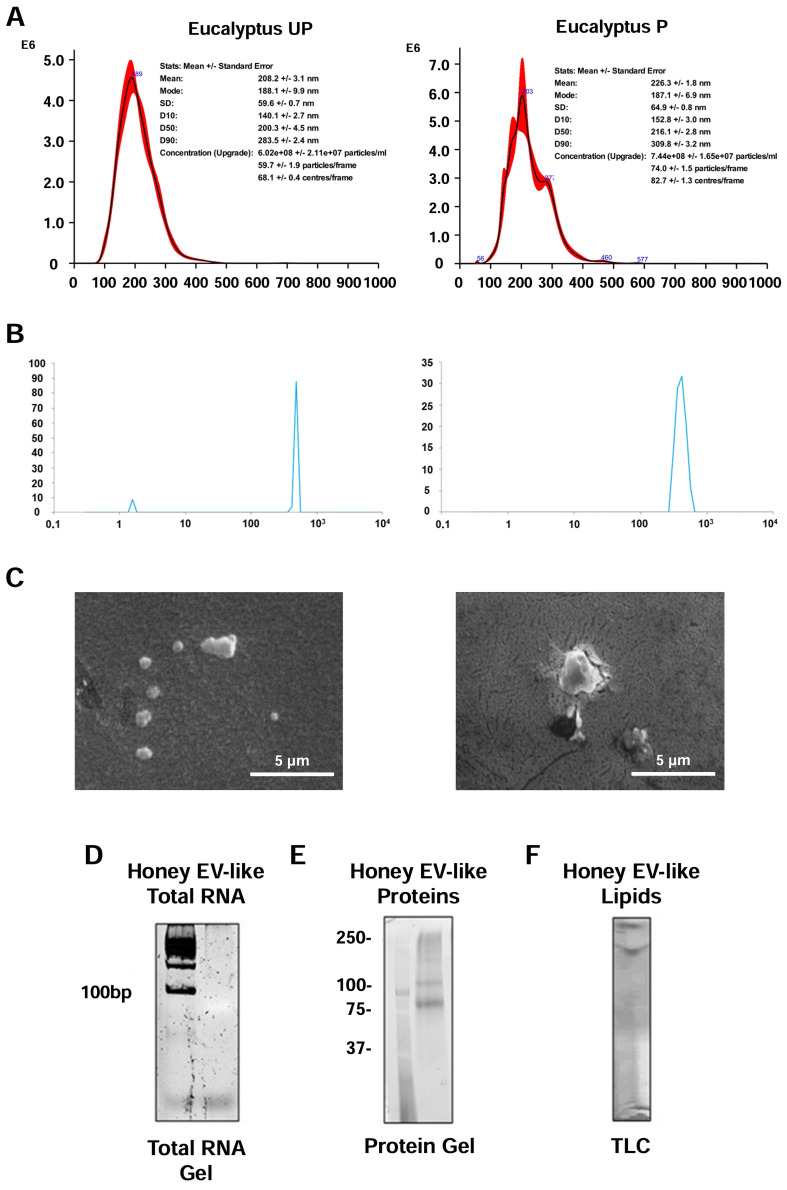
Example of characterization of EVs-like derived from Eucalyptus honey using Nanoparticle Tracking Analysis (NTA) (**A**), Dynamic Light Scattering (DLS) (**B**), and Scanning Electron Microscopy (SEM) for both unpasteurized and pasteurized samples (**C**) (Scale bar: 5 μm). (**D**) RNA gel of Honey-EV-like Total RNA. miRNAs were loaded on a 12% acrylamide gel. (**E**) Protein gel of H-VLN proteins. Proteins were run on a 4–12% Bis-Tris protein gel and visualised using the ChemiDoc Imaging System (BIO-RAD). (**F**) TLC analysis of H-VLN lipids. A TLC silica gel plate was used to run the lipids.

Raw and pasteurized samples came from the same hive and collection day and were paired by honey origin. However, pasteurization was performed by beekeepers using standard procedures. Therefore, comparisons between unpasteurized and pasteurized samples reflect commercially available products and should be interpreted as preliminary observations rather than controlled experimental evidence of heat-induced effects. EVs-like were isolated from four honey types (Eucalyptus, Orange Blossom, Chestnut, and Sulla) and biophysically characterized. NTA and DLS of Honey-EVs-like confirmed the presence of EVs-like with an average size of approximately 200 nm, consistent with the expected size range as shown in Table 2 (Supplementary Table S2). Eucalyptus EVs, like the first analyzed (Figure 1A,B). SEM imaging revealed spherical, lipid-enclosed structures typical of EVs-like, while DLS confirmed a homogeneous size distribution (Figure 1C). Moreover, particle concentrations performed on all Honey-EVs-like ranged from 7.75 × 10^7^ to 4.81 × 10^8^ particles per gram of honey, with pasteurized Eucalyptus honey exhibiting the lowest concentration. The small RNA analysis (Figure 1D) revealed a predominant signal below 100 bp, consistent with the expected size range of miRNAs and other small non-coding RNAs. Moreover, total RNA quantification from EV-like isolates yielded concentrations ranging from 4.7 to 12 ng/µL (0.29 to 0.78 µg RNA/g honey), confirming the presence of RNA within honey-derived EV-like. The EV-like characterization of other varieties is the classical markers displaying different molecular weights, as shown in Supplementary Figure S3. The honey-EVs-like proteins were subjected to SDS-PAGE, followed by STAIN-free staining using ChemiDoc Imaging System (BIO-RAD), resulting in distinct protein bands (Figure 1E). Lipid analysis by thin-layer chromatography (Figure 1F) demonstrated multiple separated lipid fractions, confirming the presence of diverse lipid species. Collectively, these data demonstrate that honey-derived EVs-like contain RNA, protein, and lipid components, confirming their structural integrity and molecular complexity.

Pasteurization was associated with differences miRNA abundance in a honey-EVs-like-type-dependent manner (Table 3). miR-1-3p generally increased after pasteurization, while miR-33a-5p decreased in most honeys. miR-34a-5p and miR-133a-3p showed increased levels in several honey types but decreased in Eucalyptus. In contrast, miR-92a-3p remained mostly stable, except for a reduction in Chestnut honey. Given that raw and pasteurized samples were paired according to honey origin, the observed differences are more likely related to thermal processing, although variability associated with beekeeper-specific pasteurization procedures cannot be completely excluded.

**Figure 2:**
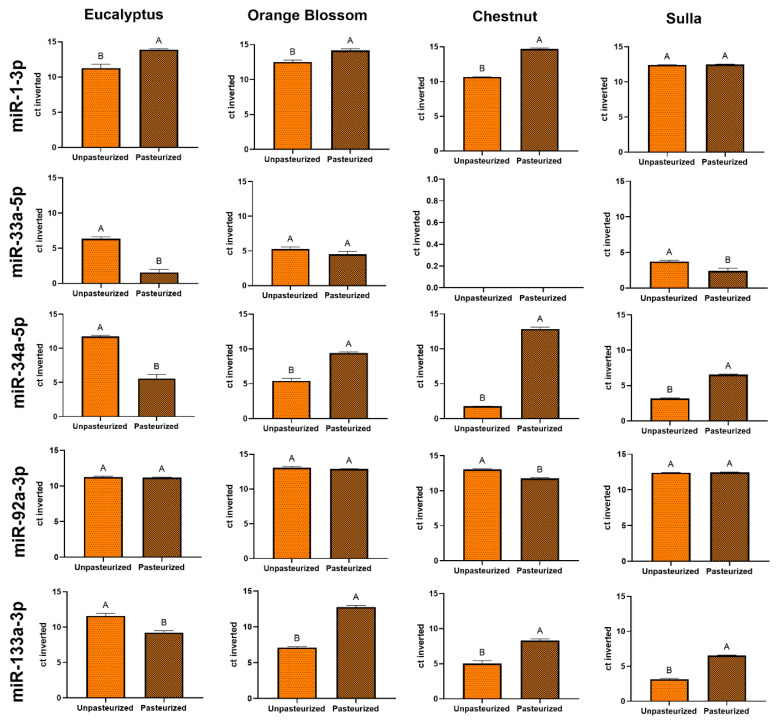
The presence of miRs was confirmed in Honey-EVs-like by RT-PCR, from the four types of honey, using three independent extractions. Inverted Ct values of miRNA detection in four types of honey: unpasteurized and pasteurized Eucalyptus, Orange Blossom, Chestnut, and Sulla. Bars represent the means of three independent biological extractions. Statistically significant differences between groups are indicated by A ≠ B (*t*-test, *p* < 0.05). Higher inverted Ct values correspond to more abundance of miRNA.

Overall, pasteurization appeared to selectively alter miRNA profiles depending on the honey variety (Figure 2). No direct conclusions regarding heat susceptibility or functional stability of specific miRNAs can be drawn from the present design.

### 2.5. Bioinformatic Prediction of Potential Functional Pathways Associated with Honey-miRNAs

**Figure 3:**
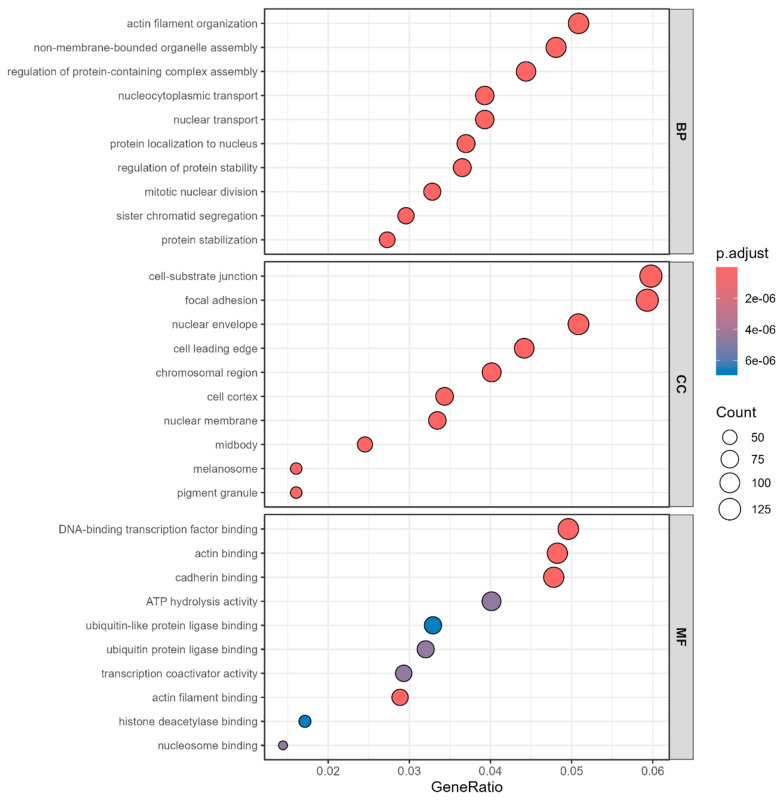
Gene Ontology (GO) analysis of signature miR targets. The term ‘GeneRatio’ indicates the number of genes from the dataset that fall into a given pathway or GO term. The dot color represents the *p*-value, using a gradient from blue (higher *p*-value, less significant) to red (lower *p*-value, more significant). The dot size corresponds to the number of genes associated with each GO term, with larger dots indicating higher gene counts.

**Figure 4:**
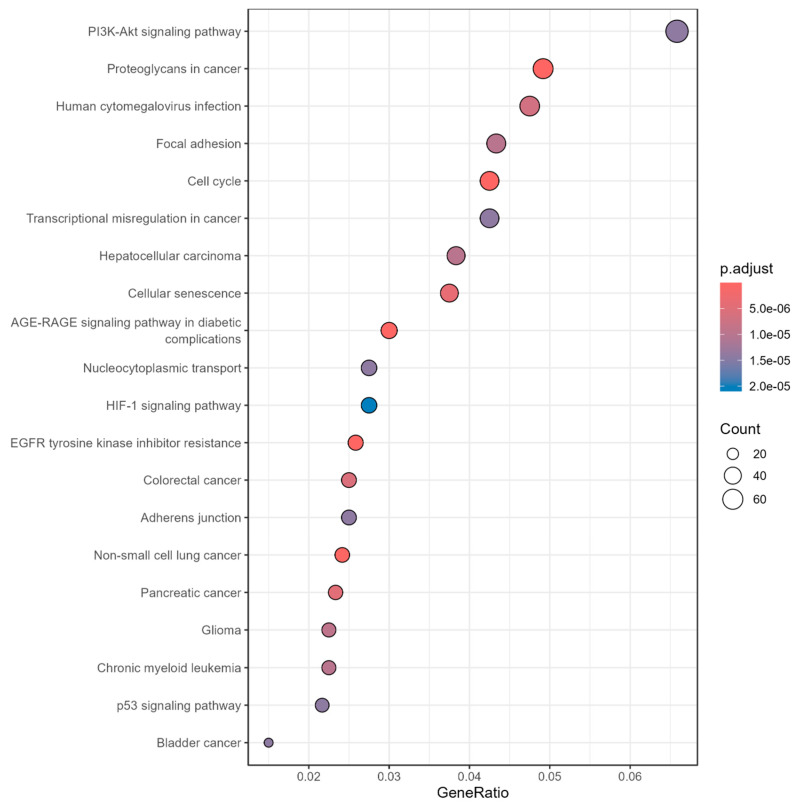
Kyoto Encyclopedia of Genes and Genomes (KEGG) Pathway Analysis. The term ‘GeneRatio’ refers to the number of genes from the dataset that fall into a specific pathway. The dot color corresponds to the *p*-value, using a gradient from blue (higher *p*-value, less significant) to red (lower *p*-value, more significant). The dot size reflects the number of genes associated with each KEGG term, with larger dots indicating higher gene counts.

**Figure 5:**
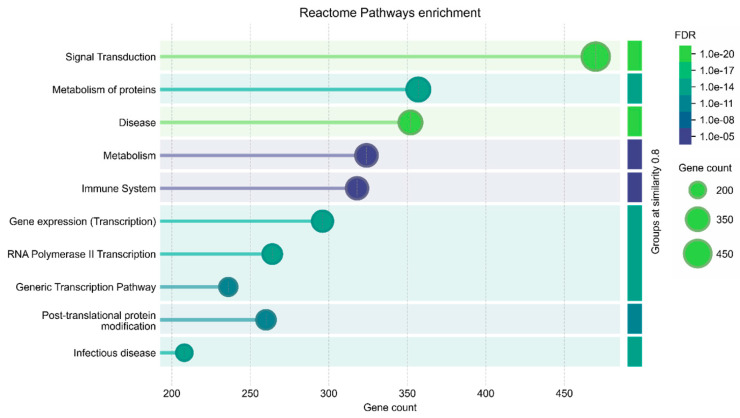
Analysis of Reactome Pathways Enrichment.

**Figure 6:**
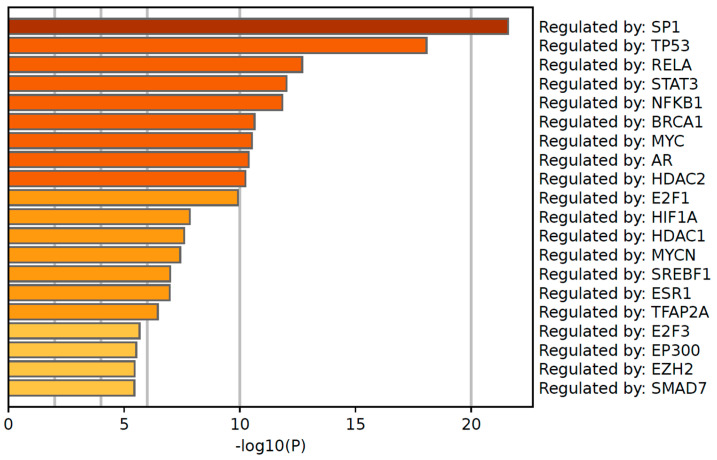
Transcription factor enrichment analysis (TRRUST) of miRNA target genes. The color of each bar represents the degree of enrichment.

To explore the potential biological relevance of the predicted miRNA targets, Gene Ontology (GO) enrichment analysis was performed (Figure 3). It is important to emphasize that the following results represent computational predictions based on target inference algorithms and enrichment databases and do not constitute experimental validation of functional effects. Within the Biological Process (BP) category, significant enrichment was observed in processes such as actin filament organization, regulation of protein-containing complex assembly, and regulation of protein stability. Other enriched processes included nucleocytoplasmic transport, nuclear transport, and mitotic nuclear division. For the Cellular Component (CC) category, enriched terms were associated with structures crucial for cell adhesion and motility, including the cell-substrate junction, focal adhesions, the cell leading edge, and the cell cortex. Additional enrichment was observed in nuclear-related compartments, including the nuclear envelope, nuclear membrane, and chromosomal regions. At the Molecular Function (MF) level, the targets were significantly enriched in actin binding, actin filament binding, and cadherin binding. Other enriched functions included DNA-binding transcription factor binding, transcription coactivator activity, and histone deacetylase binding, among others. The Kyoto Encyclopedia of Genes and Genomes (KEGG) pathways enriched for miRNA targets (Figure 4) are significantly associated with cell structure and adhesion, which are crucial for maintaining cell integrity and motility. Additionally, signaling pathways related to cell growth, differentiation, and apoptosis were prominently identified, further stressing the miRNA’s involvement in cancer-related processes. Pathways linked to immune response were also revealed, supporting the miRNA’s role in viral processes. To gain deeper insights into the interactions among miRNA targets, the genes were analyzed using the Reactome database for pathway enrichment and the STRING database for protein-protein interaction (PPI) network construction. The Reactome database maps human proteins to their corresponding molecular functions, combining expert-curated quality with robust analytical power [24]. Reactome pathway enrichment revealed strong associations with broad biological processes. The most significant pathways included Signal Transduction, Metabolism of proteins, and Disease, each supported by large gene sets (>400 genes, FDR < 1 × 10^−20^). Additional enriched categories included Metabolism and Immune System, as well as transcription-related pathways (Gene Expression, RNA Polymerase II Transcription, and Generic Transcription Pathway). More minor but significant enrichments were also observed for post-translational protein modification and infectious disease (Figure 5). Then, the STRING network consisted of 2276 nodes and 41,231 edges, forming a dense interaction landscape with an average of 37 neighbors per protein. The network had a moderate clustering coefficient (0.298) and low density (0.017), typical of biological systems. Hub proteins contributed to overall heterogeneity (1.243), while the presence of 58 connected components revealed modular organization. Notably, clusters with the highest confidence scores (Cluster 1: 106 nodes, 2736 edges; Cluster 2: 119 nodes, 1124 edges; Cluster 3: 145 nodes, 1166 edges) likely represent biologically meaningful functional modules. Due to the high number of predicted targets, PPI networks resulted in a high density. Given the density of the network, transcription factor enrichment analysis (TRRUST) was performed to identify upstream regulatory nodes. This analysis predicted convergence on master regulators including *MYC*, *TP53*, *E2F1*/*E2F3*, *STAT3*, *NFKB1*/*RELA*, and *HIF1A*, indicating that the miRNA target genes converge on key transcriptional and epigenetic control nodes rather than isolated downstream pathways (Figure 6).

Finally, cell-type enrichment analysis further suggested statistical associations between predicted target genes and signatures of specific cell populations, including gastric isthmus cells, pancreatic stromal and acinar cells, renal epithelial subsets, ovarian granulosa cells, intestinal stem cells, and several immune cell types (Supplementary Figure S4). These associations may reflect database-driven overlap with known cell-specific gene expression signatures and should be interpreted as hypothesis-generating rather than evidence of selective cellular targeting.

## 3. Discussion

Our previous research on polyfloral honey identified the presence of two widely-conserved miRNAs of the animal kingdom and established honey as a viable dietary source of miRNAs, demonstrating that a portion of its miRNA cargo survives gastrointestinal digestion [18]. However, these findings were obtained using polyfloral samples and cannot be automatically generalized to all monofloral varieties. Conserved miRNAs play critical roles in diverse biological pathways. This is because their retention and expression levels during the course of evolution may be heavily dependent on the conferred advantage offered by their regulatory activities [25,26]. The miRNAs involved in this study have identity matches ranging from 91% to 100% with the human homologs. Since mismatches in the miRNA-target pair are generally tolerated in the animal kingdom [26]. This level of identity match supports the biological plausibility of conserved target recognition, although functional activity was not assessed in the present study. We investigated the presence of conserved miRNAs in honey derived from different floral sources and evaluated the efficiency of two RNA extraction methods. The comparative analysis of RNA extraction methods revealed that the semi-automated method consistently outperformed the manual method in terms of total RNA yield and in the selective enrichment of individual miRNA levels. This improvement in yield is particularly significant for applications requiring high RNA quantities, such as downstream molecular analyses. However, the semi-automated method did not significantly enhance RNA purity, as measured by absorbance ratio at A260/A280. The relatively low A260/A280 ratios may reflect honey matrix contaminants affecting purity estimates, although target-specific TaqMan RT-qPCR confirmed suitable miRNA detection. However, the RNA yield per gram (or milliliter) of honey was within the range reported for other dietary matrices, including bovine milk fractions such as whey, extracellular vesicle-enriched preparations, and somatic cells [27,28], a well-known source of dietary RNA, but lower than that of colostrum [29]. These comparisons should be interpreted with caution, as the cited studies differ in sample matrix, isolation workflow, vesicle enrichment strategy, and RNA quantification method. Similarly, RNA concentrations in honey were lower than those found in the flowers harvested by bees [15]. This suggests that the RNA in honey may represent a subset of the RNA present in plant flowers, likely reflecting enzymatic degradation and RNAse activity during storage and processing. However, the detection of proteins and RNA expressed in the salivary glands and gut of bees in royal jelly and honey [30] indicates that honeybees may also contribute to the RNA content of honey. This may facilitate horizontal RNA transfer within bee colonies [31] and influence the RNA composition of honey. The discovery of small RNAs, such as miR-14, which are conserved across invertebrates and play essential roles in insect development, further supports this hypothesis [16]. Thus, honey serves as a unique reservoir of conserved miRNAs that resist after digestion [16,18]. In this study, we detected a panel of conserved miRNAs (miR-1-3p, miR-33a-5p, miR-34a-5p, miR-92a-3p, and miR-133a-3p) across the four monofloral honey varieties analyzed. Their presence was confirmed regardless of floral origin, suggesting that their occurrence is primarily linked to honeybee biology rather than botanical source [32,33]. Among these, miR-92a-3p showed stable detection across all honey types and was not significantly influenced by pasteurization, in agreement with our previous results in polyfloral honey [34]. Suddenly, miR-1-3p and miR-92a-3p were more abundant when extracted with the semi-automated method, particularly in Orange Blossom and Chestnut honeys, highlighting their sensitivity to the extraction approach. miR-33a-5p exhibited a general reduction following pasteurization, suggesting its heat susceptibility, while miR-34a-5p displayed variable responses depending on the honey type, with increases observed in some cultivars but decreases in Eucalyptus. Although raw and heat-treated samples originated from the same hive, pasteurization was performed according to beekeeper-specific procedures. Therefore, these results support an association between thermal processing and miRNA changes, although further studies under fully controlled pasteurization conditions are warranted. The semi-automated extraction method demonstrated superior efficiency, likely due to reduced human error, and enabled the identification of miRNAs in honey-EVs-like. The particle concentration of the EVs-like per gram of honey was lower than that reported previously [35,36,37]. The RNA concentrations in honey-EVs-like accounted for 15–27% of the total RNA in crude honey, highlighting their potential role in shuttling bioactive miRNAs, as evidenced by the presence of miRNAs in the vesicles. While vesicle association suggests a possible protective role, functional delivery and biological activity were not directly evaluated in the present study. The detection of miRNAs within EVs-like vesicles suggests that these structures may contribute to miRNA stability in honey and supports their potential relevance as natural carriers of small RNAs. However, the present study does not demonstrate that honey-derived EVs-like vesicles mediate miRNA uptake, intercellular communication, intestinal transport, or systemic delivery after ingestion. In this context, honey has been traditionally used for wound healing, with documented efficacy in treating burns, surgical wounds, and ulcers [38,39]. Its ability to promote cellular responses such as fibroblast and endothelial cell migration and proliferation enhances re-epithelialization and wound closure [40,41,42]. Bioinformatic enrichment analyses, performed using experimentally validated miRNA–target interactions, indicated that honey-miRNA targets are statistically overrepresented in biological processes related to actin cytoskeleton organization, protein stability, and transcriptional regulation, which are central to cellular homeostasis and stress adaptation [43]. Reactome pathway enrichment identified associations with signal transduction, protein metabolism, and Disease, each with substantial gene sets, suggesting convergence on core regulatory networks. Additional enriched pathways included metabolism, the immune system, and transcriptional processes, consistent with miRNA-mediated regulation of gene expression. Moreover, this analysis identified master regulators such as *MYC*, *TP53*, *E2F1*/*E2F3*, *STAT3*, *NFKB1*/*RELA*, and *HIF1A*, which govern pathways involved in proliferation, immune modulation, wound healing, and stress adaptation; reflecting predicted network convergence within the inferred target landscape rather than experimentally demonstrated transcriptional regulation. Notably, these functional predictions are consistent with previously described bioactivities attributed to honey, including immune modulation [44,45], wound healing, and antimicrobial effects [46], but they do not provide direct functional proof of these activities. A particularly intriguing observation was the enrichment of gastric isthmus cells within the cell-type signature analysis. These progenitor cells, located in the proliferative zone of gastric glands, are essential for epithelial renewal and differentiation into mucous, parietal, chief, and endocrine lineages [47]. Importantly, this association is based on computational overlap between predicted miRNA targets and known cell-type gene signatures and does not demonstrate selective targeting of gastric progenitor cells by honey-derived miRNAs. Moreover, this analysis does not account for miRNA compartmentalization within extracellular vesicles, their release during digestion, or the likelihood of uptake by gastric epithelial cells in vivo. Therefore, no gastric cell targeting or modulation of gastric epithelial renewal should be inferred without direct experimental evidence. This bioinformatic enrichment may indicate a potential area for future investigation into whether honey-associated miRNAs are linked to gene networks relevant to gastric epithelial biology [48]. The regenerative capacity of gastric isthmus cells is strongly influenced by the stromal microenvironment, particularly by fibroblasts and immune cells [49]. Cross-talk with activated fibroblasts is known to drive hyperproliferation, aberrant differentiation, and progression toward metaplasia or gastric cancer [50]. The bioavailability of dietary miRNAs remains a matter of active debate. Although some studies suggest that orally administered miRNAs may be absorbed in the stomach via SIDT1-mediated mechanisms [51] and that EVs may enhance gastrointestinal stability [52], questions persist regarding the efficiency, reproducibility, and physiological relevance of such uptake [53]. However, the present study does not provide evidence that these miRNAs survive digestion, cross the gastrointestinal barrier, reach gastric progenitor cells, or modulate gastric mucosal processes in vivo. Additional experiments aimed at quantifying honey EV-associated miRNA copy numbers following simulated digestion are currently in progress and will be required to determine their potential bioavailability. Altogether, these findings position honey not only as a reservoir of phenolic and other bioactive compounds but also as a source of EV-like-associated miRNAs whose experimentally validated target landscape intersects with pathways involved in epithelial homeostasis, immune regulation, and tissue repair. Nevertheless, these associations should be interpreted as biologically informed hypotheses pending direct functional validation in appropriate in vitro and in vivo models. A potential limitation in EV-RNA studies is RNA integrity assessment. RNA Integrity Number (RIN) analysis, developed for cellular RNA, depends on intact ribosomal RNA peaks, which are typically absent in EV-derived RNA. Because EV-RNA is enriched in small and fragmented species, RIN values are not informative in this context. Recent EV transcriptomics reviews highlight that conventional RNA quality metrics are not directly applicable to EV-associated RNA due to its intrinsic properties and low input levels [54].

## 4. Materials and Methods

### 4.1. Honey Samples

In October 2023, pasteurized and unpasteurized samples of four different types of honey (Eucalyptus, Orange Blossom, Chestnut, and Sulla) were collected from beekeepers in central and northwestern Calabria, Italy. Honey samples were obtained directly from local beekeepers. For each honey type, samples were collected from the same hive. Thus, samples were paired by origin, although pasteurization was performed by beekeepers using standard procedures (60 °C for 30 min) not controlled by the authors.

### 4.2. Honey-Derived Extracellular Vesicles-like Isolation and Characterization

Each honey sample was diluted 1:1 in sterile phosphate-buffered saline (PBS), filtered through a 0.45 µm membrane, and stored at room temperature prior to RNA extraction and EV isolation. The EVs-like were isolated using ultracentrifugation at 200,000× *g* for 1 h at 4 °C. Ultracentrifugation was performed using an MLA-50 rotor (Beckman Coulter, Brea, CA, USA).

The resulting EV-like pellet was carefully washed with sterile PBS and then resuspended in 100 µL of PBS for subsequent analysis. The EVs-like were characterized for particle concentration and size distribution using Nanoparticle Tracking Analysis (NTA; NanoSight NS 3000, Malvern Instruments, Malvern, UK) and Dynamic Light Scattering (DLS; Malvern Zetasizer Nano ZS, Malvern Instruments, Malvern, UK). EVs-like were diluted 1:100 in PBS and analyzed at 25 °C. Data were processed using NTA 3.2 software (Malvern Instruments Ltd., Malvern, UK) for NTA measurements and Zetasizer v.7.10 software (Malvern Instruments) for DLS analysis. EV-like size and morphology were analyzed using Scanning Electron Microscopy (SEM) (Thermo Fisher Scientific, Waltham, MA, USA). A 1 µL sample of vesicles was deposited onto an SEM stub pre-coated with a glass slide. To minimize charging effects and facilitate high-resolution imaging of non-conductive samples, a gold sputter coating was applied. The images were captured using a Phenom™ ProX Desktop SEM (Thermo Scientific™, Waltham, MA, USA) with the following system parameters: accelerating voltage of 15 kV, low beam intensity, and SED detector.

EVs were isolated from two different batches of each type of honey (Eucalyptus, Orange Blossom, Chestnut, and Sulla). The EVs-like, isolated according to a standardized protocol, were used for characterization analyses. All experiments were performed using multiple biological and technical replicates to ensure the reproducibility and reliability of the results.

### 4.3. Honey-Derived Extracellular Vesicles-like Protein Gel Electrophoresis

The Honey-Derived EVs were lysed in RIPA 5× for 30 min on ice and subsequently centrifuged for 10 min at 14,000 rpm. 30 µg of samples were re-suspended in 1× Laemmli buffer and heated at 95 °C for 5 min. Gel electrophoresis in reducing conditions (SDS-PAGE) was performed on honey-EV proteins using commercially available 4–12% Bis-Tris polyacrylamide electrophoresis gels (Mini-PROTEAN^®^TGX Stain-Free, BIO-RAD, Hercules, CA, USA). Protein bands were visualized using the ChemiDoc Imaging System (BIO-RAD) and analyzed with Image Lab™ Software version 5.2.1 (BIO-RAD).

### 4.4. Lipids Extraction and Characterization from Honey-Derived Extracellular Vesicles-like

Lipids were purified from Honey-Derived EVs-like using the Folch method [35,55] and run on a silica gel TLC plate (EMD Millipore, Burlington, MA, USA) using a solvent mixture of chloroform/methanol/acetic acid (190:9:1, Sigma, St. Louis, MO, USA). As previously described by Chen X. et al., the extract was stained with 10% CuSO_4_ in 8% phosphoric acid (Sigma).

### 4.5. Total RNA Extraction from Honey

Total RNA was extracted from honey using two distinct methods: (i) The manual column-based method, in which RNA extraction was performed using the miRNeasy serum/plasma Kit (Qiagen, Hilden, Germany). For all samples, 280 mg of honey was mixed by vortexing with 200 µL of RNase-free water. The mixture was then incubated at room temperature for 5 min with 1 mL of lysis reagent. Following this, the entire volume was processed according to the manufacturer’s protocol; and (ii) the semi-automated RNA isolation, which was carried out using the MagMAX mirVana Total RNA Isolation Kit (Thermo Fisher Scientific, Waltham, MA, USA) on the KingFisher Duo Prime Magnetic Particle Processor (Thermo Fisher Scientific, Waltham, MA, USA). This system leverages magnetic bead technology for efficient and reproducible RNA extraction, minimizing manual handling and reducing the risk of contamination. Either here, 280 mg of monofloral honey was diluted with 200 µL of RNase-free water. A 100 µL aliquot of the diluted honey or its EVs-like was transferred to a 96-deep-well plate, followed by the addition of 45 µL of Digestion Buffer and 5 µL of Proteinase K. Subsequent steps were performed according to the manufacturer’s instructions, which include magnetic bead-based RNA capture, washing, and elution. RNA concentrations and quality were measured using a NanoDrop One spectrophotometer (Thermo Fisher Scientific, Waltham, MA, USA). RNA integrity and small RNA enrichment were assessed by electrophoresis on a 12% polyacrylamide gel.

### 4.6. Reverse Transcription and RT-PCR

Complementary DNA (cDNA) was synthesized using the TaqMan Advanced miRNA cDNA Kit (Thermo Fisher Scientific, Waltham, MA, USA) with sequence-specific primers obtained from the same manufacturer. All cDNA synthesis reactions were carried out in an Applied Biosystems™ 2720 Thermal Cycler (Applied Biosystems, Madrid, Spain). After normalizing the isolated RNA samples, 10 ng of RNA was used for the poly(A) tailing reaction. The reaction products were then sequentially processed through adaptor ligation, reverse transcription, and cDNA synthesis steps, according to the manufacturer’s protocol. A reaction mixture was prepared using 5 µL of diluted cDNA (1:10 dilution with RNase-free water), 10 µL of 2× TaqMan Advanced Master Mix, 4 µL of RNase-free water, and 1 µL of 20× TaqMan Advanced miRNA. Assay containing the specific primers (details in Supplementary Material Table S1). A synthetic non-mammalian miRNA (cel-miR-39) was added to each sample prior to RNA extraction as an exogenous spike-in to monitor extraction efficiency and technical variability. The reaction setup was performed in a 96-well plate, and the plate was centrifuged at 4000 rpm for 5 min to ensure all contents were collected at the bottom of the wells, minimizing variability. Each biological sample was analyzed in technical triplicate to ensure statistical robustness and reproducibility, adhering to the Minimum Information for Publication of Quantitative Real-Time PCR Experiments (MIQE) guidelines [56]. Amplification was performed using a Quant Studio 5 Real-Time PCR System (Applied Biosystems, Madrid, Spain) under fast-cycling conditions with a comparative Ct (ΔΔCt) analysis mode. The thermal cycling protocol consisted of an initial enzyme activation step at 95 °C for 20 s, followed by 40 cycles of denaturation at 95 °C for 1 s and annealing/extension at 60 °C for 20 s. Given the lack of validated endogenous reference controls for honey-derived EV-like preparations, no endogenous normalization was applied. RT-qPCR was used to assess selected miRNA detectability, expressed as inverted 40−Ct values, with undetermined considered negative. Thus, these values should be interpreted as detection-based RT-qPCR readouts rather than fully normalized expression data [18,57].

### 4.7. Bioinformatic Analyses

The miRBase database was utilized to identify sequence homology between human miRNAs and their honeybee counterparts, providing insights into the evolutionary conservation and potential cross-species functionality of miRNAs detected in honey. Next, the mRNA targets were validated using multimiR v2.4 (accessed 21 August 2025) [58], with filtered results based on a *p*-value ≤ 0.01. FDR correction was applied to control for the false discovery rate and the presence of at least two databases. To further elucidate the biological roles of these targets, Gene Ontology (GO) and Kyoto Encyclopedia of Genes and Genomes (KEGG) pathway enrichment analyses were performed using the ClusterProfiler package in R (RStudio version, v4.2.2, 6 January 2023) [59]. Reactome pathways networks were constructed using the STRING online tool (https://string-db.org/, accessed 23 August 2025) with a medium confidence of 0.4 and 5% FDR [60]. PPI networks were constructed using the STRING app on Cytoscape v. 3.9.1 [61]. To identify densely connected regions within these networks, clustering was performed using the Molecular Complex Detection (MCODE) algorithm version 2.0.2 [62], which highlights potential functional modules or protein complexes. Finally, transcription factor enrichment analysis (TRRUST) and Cell Type Signatures were created with Metascape v3.5 [63]. Terms with a *p*-value < 0.01, a minimum count of 3, and an enrichment factor > 1.5 (the enrichment factor is the ratio between the observed counts and the counts expected by chance). These parameters correspond to commonly applied default settings in the platform and were chosen to balance statistical stringency with biological interpretability, reducing false positives while retaining pathways supported by multiple genes.

### 4.8. Statistical Analysis

Data are reported as mean ± SD (or SEM) of biological replicates. One-way or two-way analysis of variance (ANOVA) followed by Dunnett’s multiple comparison test was used to generate statistical analysis using the GraphPad Prism program (version 10.2.3); *p* values < 0.05 were considered statistically significant. Additionally, pathway enrichment results were visualized using the SRplot dot plot tool (http://www.bioinformatics.com.cn/srplot, accessed on 7 June 2026) [64].

## 5. Conclusions

This study identifies potential regulatory pathways associated with conserved miRNAs packaged within honey-derived EVs-like through bioinformatic analyses, establishing a hypothesis-generating framework for future validation of their roles in immune modulation and epithelial repair. Importantly, the apparent protection of miRNAs within these preparations should be interpreted as inferred from their detectability rather than demonstrated by direct degradation assays. Collectively, these findings support further investigation into the biological relevance of honey-derived EV-like miRNA cargo and its prospective applications in biomedicine, while future preclinical studies should include appropriate controls for endogenous miRNA background in recipient tissues, as well as experimental validation of miRNA stability, uptake, and functional activity.

## Figures and Tables

**Table 1:** Total RNA yield and purity according to the RNA extraction method and honey variety.

Sample	Pasteurization	Manual Extraction		Semi-Automated Extraction	
		**μg RNA/g** **Sample**	**A_260/280_**	**μg RNA/g** **Sample**	**A_260/280_**
**Eucalyptus**	No	0.51	1.60	2.86	1.71
	Yes	0.65	1.45	2.57	1.68
**Orange Blossom**	No	0.95	1.46	1.93	1.47
	Yes	0.75	1.47	1.61	1.43
**Chestnut**	No	0.45	1.55	3.46	1.50
	Yes	0.49	1.71	2.30	1.66
**Sulla**	No	0.54	1.51	2.59	1.57
	Yes	0.77	1.51	3.38	1.81

**Table 2:** Characterization of Honey-EV like.

Sample	Pasteurization	Concentration (Particles/g Honey)	Diameter (nm)	µg RNA/g Honey
**Eucalyptus**	No	7.75 × 10^7^ ± 2.72 × 10^6^	208.2	0.779
	Yes	8.93 × 10^7^ ± 1.98 × 10^6^	226.3	0.480
**Orange Blossom**	No	7.89 × 10^8^ ± 3.33 × 10^7^	245.2	0.462
	Yes	2.99 × 10^9^ ± 2.10 × 10^8^	217.5	0.287
**Chestnut**	No	9.42 × 10^8^ ± 1.92 × 10^7^	214.2	0.516
	Yes	4.81 × 10^9^ ± 5.59 × 10^8^	233.7	0.291
**Sulla**	No	2.36 × 10^9^ ± 1.65 × 10^8^	205.1	0.617
	Yes	2.68 × 10^9^ ± 1.71 × 10^8^	207.9	0.515

Data represented as Mean ± SD. The diameter was determined by NTA. RNA was extracted with the semi-automated method.

**Table 3:** Effects of pasteurization on miRNA presence in different honey types.

miRNA	Eucalyptus	Orange Blossom	Chestnut	Sulla
**miR-1-3p**	↑	↑	↑	=
**miR-33a-5p**	↓	↓	Ø	↓
**miR-34a-5p**	↓	↑	↑	↑
**miR-92a-3p**	=	=	↓	=
**miR-133a-3p**	↓	↑	↑	↑

Arrows indicate the direction of significant changes (*p* < 0.05) after pasteurization (↑ increase; ↓ decrease; = no change; Ø not detectable). Specifically, miR-1-3p showed a significant increase in pasteurized Eucalyptus, Orange Blossom, and Chestnut samples, whereas no significant differences were observed in Sulla honey. miR-33a-5p was significantly reduced in pasteurized Eucalyptus and Sulla samples, with no significant changes in orange blossom, and not detected in chestnut. For miR-34-5p, a significant decrease was observed in Eucalyptus, while a significant increase was detected in Orange Blossom, Chestnut, and Sulla. miR-92a-3p remained largely unchanged across most honey types, except for a slight reduction in Chestnut. Finally, miR-133a-3p displayed a differential pattern, with a significant decrease in Eucalyptus and significant increases in Orange Blossom, Chestnut, and Sulla. Data based on the inverted Ct.

## Data Availability

The original contributions presented in this study are included in the article/Supplementary Material. Further inquiries can be directed to the corresponding author.

## Supplementary Materials

The following supporting information can be downloaded at: https://www.mdpi.com/article/10.3390/ijms27125297/s1.
