# Supplementary material for: Extracellular Vesicle-like Associated microRNAs in Monofloral Honeys: Molecular Characterization and Functional Pathways
Source: Int J Mol Sci. 2026 Jun 11;27(12):5297. doi: 10.3390/ijms27125297 (PMC13300486; doi:10.3390/ijms27125297)
Supplement: Supplementary file 1 [file ijms-27-05297-s001.zip › ijms-4344228-supplementary.pdf]

Supplementary Material

Table S1. Taqman™ Probes ID, miRBase Annotation and mature sequence.

#	Probe ID	miRBase Annotation	Mature Sequence (5'-3')
**1**	478293_mir	let-7a-5p	UGAGGUAGUAGGUUGUAUAGUU
**2**	477820_mir	miR-1-3p	UGGAAUGUAAAGAAGUAUGUAU
**3**	483061_mir	miR-7-5p	UGGAAGACUAGUGAUUUUGUUGUU
**4**	479241_mir	miR-10a-5p	UACCCUGUAGAUCCGAAUUUGUG
**5**	478347_mir	miR-33a-5p	GUGCAUUGUAGUUGCAUUGCA
**6**	478048_mir	miR-34a-5p	UGGCAGUGUCUUAGCUGGUUGU
**7**	477827_mir	miR-92a-3p	UAUUGCACUUGUCCCGGCCUGU
**8**	477885_mir	miR-125b-5p	UCCCUGAGACCCUAACUUGUGA
**9**	478511_mir	miR-133a-3p	UUUGGUCCCCUUCAACCAGCUG
**10**	478293_mir	cel-miR-39-3p	UCACCGGGUGUAAAUCAGCUUG

Table S2. Polydispersity Index (PDI) of honey-derived vesicle preparations.
Polydispersity Index (PDI) values obtained by Dynamic Light Scattering (DLS) analysis for all analysed samples.

Polydispersity Index (PDI)		
	**NP**	**P**
Eucalyptus	0.75	0.24
Chestnut	0.36	0.39
Orange blossom	0.5	0.38
Sulla (Hedysarum coronarium)	0.52	0.23

Let-7a-5p

```
Identity: 100.00%  Query coverage: 86.36%
Target coverage: 90.48%  Gaps: 0.00%
Query  2 GAGGUAGUAGGUUGUAUAG 20
         |||||||||||||||||||
Sbjct  2 GAGGUAGUAGGUUGUAUAG 20
```

miR-1-3p

```
Identity: 100.00%  Query coverage: 86.36%
Target coverage: 86.36%  Gaps: 0.00%
Query  1 UGGAAUGUAAAGAAGUAUG 19
         |||||||||||||||||||
Sbjct  1 UGGAAUGUAAAGAAGUAUG 19
```

miR-7-5p

```
Identity: 100.00%  Query coverage: 87.50%
Target coverage: 25.29%  Gaps: 0.00%
Query  2 GGAAGACUAGUGAUUUUGUUG 22
         |||||||||||||||||||||
Sbjct  2 GGAAGACUAGUGAUUUUGUUG 22
```

miR-10a-5p

```
Identity: 100.00%  Query coverage: 91.30%
Target coverage: 100.00%  Gaps: 0.00%
Query  2 ACCCUGUAGAUCCGAAUUUGU 22
         |||||||||||||||||||||
Sbjct  1 ACCCUGUAGAUCCGAAUUUGU 21
```

miR-33a-5p

```
Identity: 100.00%  Query coverage: 85.71%
Target coverage: 21.00%  Gaps: 0.00%
Query  1 GUGCAUUGUAGUUGCAUU 18
         ||||||||||||||||||
Sbjct  1 GUGCAUUGUAGUUGCAUU 18
```

miR-34a-5p

```
Identity: 90.00%  Query coverage: 86.36%
Target coverage: 90.91%  Gaps: 5.00%
Query  2 GGCAGUGUC-UUAGCUGGUU 20
         ||||||||  ||||||||||
Sbjct  2 GGCAGUGUUGUUAGCUGGUU 21
```

miR-92a-3p

```
Identity: 95.00%  Query coverage: 90.91%
Target coverage: 95.24%  Gaps: 0.00%
Query  2 AUUGCACUUGUCCCGGCCUG 21
         |||||||||||||||||||
Sbjct  1 AUUGCACUUGUCCCGGCCUA 20
```

miR-125a-5p

```
Identity: 86.36%  Query coverage: 91.67%
Target coverage: 90.91%  Gaps: 9.09%
Query  2 CCCUGAGACCCUUUAACCUGUG 23
         ||||||||||||  ||| ||||
Sbjct  2 CCCUGAGACCCU--AACUUGUG 21
```

miR-133a-3p

```
Identity: 100.00%  Query coverage: 95.45%
Target coverage: 95.45%  Gaps: 0.00%
Query  2 UUGGUCCCCUUCAACCAGCUG 22
         |||||||||||||||||||||
Sbjct  1 UUGGUCCCCUUCAACCAGCUG 21
```

Figure S1. Alignment of human miRNA signature with Apis mellifera Homolog.

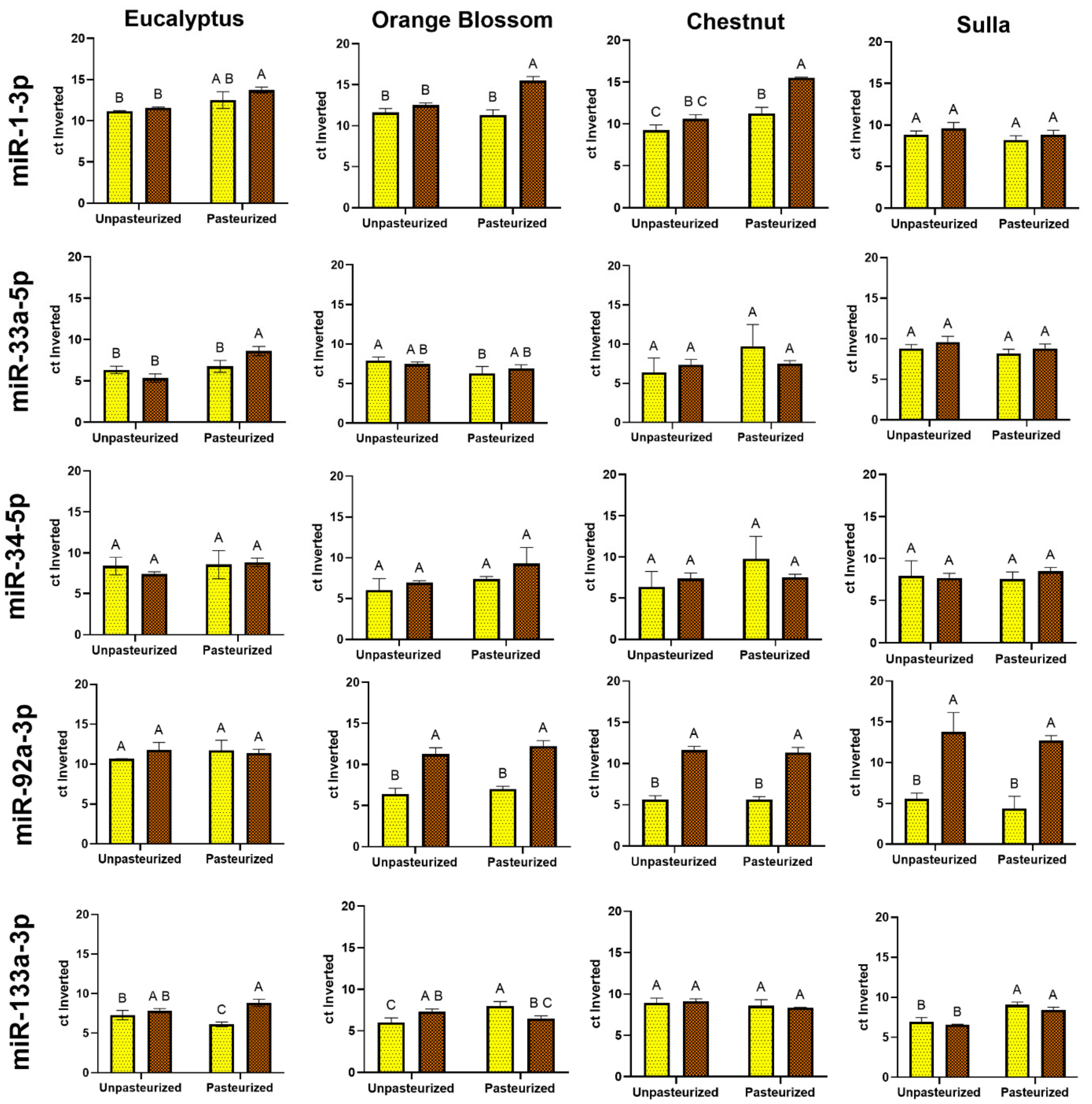

Figure S2. Inverted Ct values of miRNAs detection in four types of honey, unpasteurized (UP) and pasteurized (P)—Eucalyptus, Orange Blossom, Chestnut, and Sulla—were compared between manual and semi-automated RNA extraction methods under both unpasteurized and pasteurized conditions. Bars represent the means of three independent biological extractions. Statistically significant differences between groups are indicated by A≠B ≠C (2-Way ANOVA, $P < 0.05$). Higher inverted Ct values correspond to a greater presence of miRNA. Light yellow: Manual extraction; Dark yellow: semi-automated extraction.

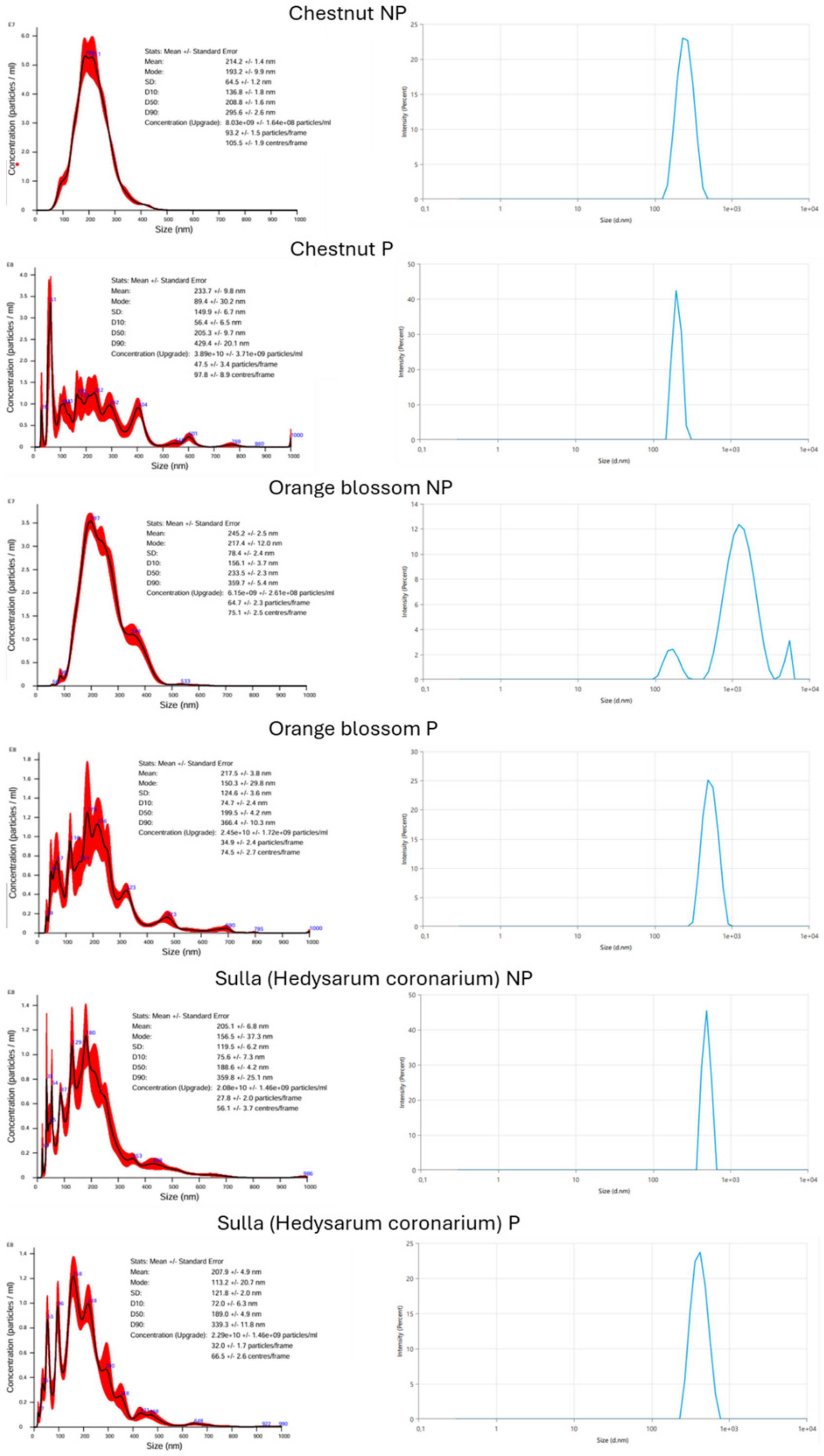

Figure S3. Characterization of EVs derived from Chestnut, Orange blossom and Sulla (Hedysarum coronarium) honeys. Nanoparticle Tracking Analysis (NTA) and Dynamic Light Scattering (DLS) for both unpasteurized and pasteurized samples.

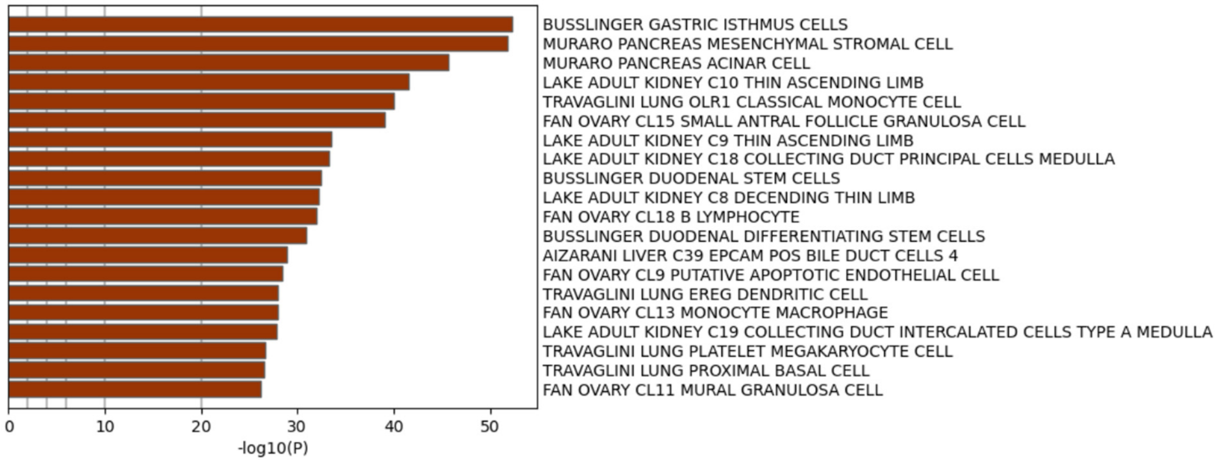

Figure S4. Summary of enrichment analysis in Cell Type Signatures**.**
